# Evaluating the Effectiveness of Digital Content Marketing Under Mixed Reality Training Platform on the Online Purchase Intention

**DOI:** 10.3389/fpsyg.2022.881019

**Published:** 2022-06-30

**Authors:** C. H. Li, O. L. K. Chan, Y. T. Chow, Xiangying Zhang, P. S. Tong, S. P. Li, H. Y. Ng, K. L. Keung

**Affiliations:** ^1^School of Science and Technology, Hong Kong Metropolitan University, Hong Kong, Hong Kong SAR, China; ^2^Division of Business and Hospitality Management, College of Professional and Continuing Education, The Hong Kong Polytechnic University, Hong Kong, Hong Kong SAR, China; ^3^Institute of Industrial Engineering, School of Mechanical Engineering, Zhejiang University, Hangzhou, China; ^4^Re-Industrialisation, Hong Kong Science and Technology Parks Cooperation, Hong Kong, Hong Kong SAR, China; ^5^Department of Industrial and Systems Engineering, The Hong Kong Polytechnic University, Hung Hom, China

**Keywords:** digital content marketing, social media marketing, marketing, customer engagement, structural equation modeling, customer loyalty, mixed reality

## Abstract

The purpose of this research is to investigate the effectiveness of Digital Content Marketing (DCM) on a Mixed Reality (MR) training platform environment with the consideration of online purchase intention (OPI) through social media. E-commerce today encounters several common issues that cause customers to have reservations to purchase online. With the absence of physical contact points, customers often perceive more risks when making purchase decisions. Furthermore, online retailers often find it hard to engage customers and develop long-term relationships. In this research, a Structural Equation Model (SEM) is proposed to examine the efficacy of DCM from both immediate and long-term OPI. The results examine whether adopting DCM on an MR training platform environment through social media brings positive results in OPI. Empirical research was carried out through online questionnaires collected in 2021 and 2022. A total of 374 questionnaires were qualified for data analysis in this study, conducted with IBM SPSS and AMOS. The results imply that DCM is critical to stimulating both immediate and long-term OPI. The immediate OPI is positively affected by increasing perceived value through MR in DCM. Regarding the long-term OPI, increased customer engagement with DCM under MR environment can cultivate brand trust and significantly affect the long-term OPI.

## Introduction

Marketing strategy is crucial in a business plan. Apart from triggering short-term sales, it determines corporate image and acts as a bridge between customers and sellers so that both parties can communicate and build a relationship. Advertising draws people’s attention to a brand’s message on a product, service, information, or idea. Traditional advertising is often displayed on billboards, external walls of buildings, magazines, newspapers, leaflets, and TV commercials. Digital Content Marketing (DCM) is a way of marketing by creating and distributing content online to deliver valuable and engaging content to customers (Rowley, 2008; Holliman, 2014). Since the last decade, digital advertising has become much more critical than before as it is more affordable and can reach larger audiences. 5G networking is being adopted worldwide increasingly so the access speed of digital content can be instantaneous with the aid of the Internet of Things (Keung et al., 2018, 2020, 2021, 2022a,b; Lee et al., 2018b; Liu et al., 2019; Li et al., 2021b,c,d; Xia et al., 2021; Zheng et al., 2021; Fan et al., 2022; Zhang et al., 2022). DCM aims to build a close connection with customers through continuous conversation to convince their leads over time. A company can show its expertise and strengths through delivered content so that customers may be convinced that its offering is valuable and worth purchasing (Hollebeek and Macky, 2019) and can realize more on their actual needs of a product (Thomson and Laing, 2003; Grant et al., 2007; Dawes and Nenycz-Thiel, 2014). Consequently, DCM can reach more potential customers, boost online purchase intention (OPI), and retain customer loyalty (Rowley, 2008; Holliman, 2014). Considering the online seller’s perspective, a reliable and valuable e-commerce environment can retain customers, reach higher customer retention and boost sales, as the traditional marketing industry is progressively burdensome and cost-ineffective (Khan and Siddiqui, 2013).

Meanwhile, misleading advertising becomes an increasing concern for digital marketing, as customers cannot do a hands-on inspection to ensure product quality. It may create false belief in the expected product performance, which varies from the actual product information to deceit, hidden contracts, fees, inexistent benefits, and exaggeration (Sharma and Chander, 2011; Kumar and Gunaseelan, 2016). Sharma and Chander (2011) found that even though people understood the existence of misleading advertising, they could not always distinguish the trustfulness and authenticity of the product. This phenomenon may eventually affect customers’ decision-making on whether to repurchase a product or not (Jacoby et al., 1982). Compared with shopping in a physical store, consumers perceive more risk of misleading and deceptive practices when online shopping (Ahmad et al., 2016; Consumer Council, 2016). In the long term, it will affect the customer benefit and satisfaction and lead to low OPI and consumer confidence in the future (Hollebeek et al., 2016; Marbach et al., 2016; Azer and Alexander, 2020).

The second issue in digital marketing is that its payoff is not straightly associated with an advertisement’s spending. Paid advertisements are too intrusive, so that customers may feel annoyed and ignore them; therefore, the effectiveness of paid advertisements is likely to become minimal (Barreto, 2013). Tudoran (2019) found that the majority of people had a negative perception of intrusive marketing, as it is always one-way and irrelevant. Some responses received by Truong and Simmons (2010) stated that the responders were rarely concerned with banner advertisements and felt annoyed when they were searching for other valuable information. Internet users can access thousands of information every day, distinguishing the authenticity of an advertisement’s information. Most content is provided free of charge or at a low cost since web 3.0 started. Therefore, selling a product to Internet users becomes more challenging through traditional marketing strategies. If the contents of an advertisement bores its audience or fails to give confidence to the audience, most of them will close the pop-up commercials as soon as possible and even install pop-up blockers. In contrast, Internet users today appreciate advertising messages that are customized, valuable, and under control (Xu et al., 2020; Ali et al., 2021; Liu et al., 2021; Siddique et al., 2021; Jamil et al., 2022; Shiyong et al., 2022; Wang et al., 2022).

Mixed Reality (MR) is an integration of augmented and virtual reality. As the latest immersive technology among the three, MR is involved in functional mockups, military training, medical care, and many other fields. It combines digital and real worlds to unblock the linkage between human, computer, and environment interaction. Users can communicate with digital items placed in the physical world in real-time (Liu et al., 2017; Li et al., 2021d). The virtual objects will be able to respond to users when they are equipped with the necessary equipment. For example, the MR Headset is adopted to deliver a credible and three-dimensional mixed-reality experience. To enhance the overall customer experience for online shopping, MR technologies could be adopted to create technology-enhanced customer experiences (Alcañiz et al., 2019; Flavián et al., 2019). Castillo and Bigne (2021) proposed a model that extends the technology acceptance model by introducing factors that affect the consumers’ acceptance of augmented reality (AR) self-service technologies, providing new understandings for retailers on the adoption of AR at the point of sale. Wedel et al. (2020) proposed a conceptual framework for VR/AR research in consumer marketing that intensifies around customer experiences provided by VR/AR implementation along the consumer journey and the effectiveness of such VR/AR implementation toward consumer marketing. Alcañiz et al. (2019) further extended the VR in marketing and proposed a research agenda for VR in marketing. However, the current literature has not considered the MR-based platform for the DCM, primarily through social media. The current hypothesis models have not been tested under an MR-based platform for DCM. When compared to traditional DCM-based research with questionnaires, we further extend the scope of the field for exploring the DCM strategies that will affect the immediate and long-term OPI under the MR training platform.

Besides achieving the immediate purchase intention (in terms of product/service) by delivering helpful content to the audience, DCM can cultivate trust and customer loyalty by customer engagement. An effective way to retain customer loyalty is to build the relationship through many conversations and deliver valuable and accurate information to the audience. This way, companies can affect the mindset of the audiences over a long duration of time. Compared to traditional marketing, digital marketing technology is more affordable and easy to use. With DCM, even the SMEs can achieve an effective marketing campaign and access to their targeted customer with great content. Li et al. (2002) pointed out that reducing intrusiveness has a significant positive impact on advertising effectiveness and customer engagement. Therefore, e-commerce can capture their customer’s favor, a massive amount of data during the conversation and provide a customized product. During the first one and half years, a paid search campaign is effective. However, leads from paid search campaigns are constant, while content marketing has exponential growth. Content marketing can produce three times more than a paid search campaign in the last month of the third year. Thus, the SMEs should not give up on developing DCM, and they cannot initially observe a decisive result. The long-tail effect of DCM under an MR-based training platform will surprise everyone, as it requires time to have an exponential effect. Social media networks are the most popular way people are willing to grasp information. Users are willing to search, follow, like, and comment on a post they are interested in; hence, user-generated content can be developed. Therefore, DCM seems able to present selling messages to their targeted customer effectively, avoiding the issues of traditional paid advertisements, and at the same time is price valued. With extraordinary performance, DCM can achieve a company’s marketing objectives at a low cost. SMEs should involve the DCM in their marketing activities. The aims of this paper include:

- To evaluate the effectiveness of DCM through social media under the MR-based platform to immediate and long-term OPI.
- To evaluate the mediating effect of perceived value, customer engagement, and brand trust.
- To discuss the managerial implications of using DCM in an MR-based environment.

Adopting DCM with social media under an MR-based environment provides valuable and engaging content to raise immediate OPI and enables customer engagement to build trust and long-term OPI. This study develops a hypothesis model of DCM under an MR-based platform to conduct the empirical study for evaluating the effectiveness of DCM on OPI through social media by using Structural Equation Modeling (SEM). The confirmatory factor analysis (CFA) is adopted to test the developed conceptual model. The perceptions of Hong Kong citizens, active social media users, on DCM are captured from the questionnaires. The analysis is concentrated on DCM on Instagram, a popular online social media platform in Hong Kong. Section “Literature Review and Hypothesis” presents the theoretical background and a hypothesis model of the research. Section “Methodology” presents the research methodology. CFA is performed after the hypothesis model has been developed. The perception of Hong Kong citizens, who are active social media users, on DCM is captured by questionnaire. The results and discussion of the effectiveness of DCM are presented in Section “Data Analysis and Results,” respectively. The survey results provide theoretical and managerial implications in Section “Discussion.” Conclusion, limitations, and future research are discussed in the below section.

## Literature Review and Hypothesis

Digital marketing is the component of marketing that utilizes the Internet and online-based digital technologies to promote products and services, such as desktop computers and mobile phones. Digital marketing campaigns have become prevalent as the number of digital platforms and e-commerce platforms increase, and as people discover that online shopping is more convenient and time-efficient. It employs combinations of search engine optimization (SEO), search engine marketing, content marketing, influencer marketing, data-driven marketing, e-commerce marketing, social media marketing (SMM), direct email marketing, and advertising (Wang and McCarthy, 2020; Bowden and Mirzaei, 2021; Mathew and Soliman, 2021; Yaghtin et al., 2021).

Social Media, built on Web 2.0 technology, allows users to share, discuss, and exchange content. It is open, accessible, and content-based so users can access the content on either technology, time, geographical, ability, or identity. Users can share content instantaneously and access the audience anytime and anywhere. There is an increasing number of online sellers advertising and selling their products on social media. The above strategy is called SMM. There is nearly no additional cost to e-commerce, and sellers will face a minimal entry barrier. Sellers can also understand their customers through direct conversation and interaction, such as discovering and sharing product information and delivering valuable opinions. Therefore, the sellers can target the customers who have an enormous willingness to buy and suggest appropriate products to them, and finally, customers can make purchase decisions.

Customer engagement can be cultivated by participating in commercial activities, marketing campaigns, and interaction, including viewing, liking, commenting, and sharing the content (Amblee and Bui, 2011). Positive electronic word-of-mouth (eWOM) can be created when the posts have numerous likes with encouraging comments. Potential customers will have a more favorable attitude and confidence toward the product. It can raise trust in online sellers and boost purchase intention (Hajli, 2014).

Instagram, a popular social media platform in Hong Kong, is mainly a visual-based photo and video-sharing social networking platform. By sharing products’ information, online sellers can attract potential consumers and drive consumer engagement through the photography-based function of Instagram (Bergström and Bäckman, 2013). A simple and most crucial rule to gain advantage in the Instagram algorithm is to generate quality content and deliver it to users. People have found that when an account with more than 5,000 followers creates 5–6 posts every day, Instagram will deliver its posts to other users who have not followed the account. Moreover, a brand can gain an advantage with UGC, which is the content developed or created by general users. For example, “like,” share, and comment can increase attention and browse traffic, tagged posts can be found in the brand’s profile, and users can generate more quality content. With UGC, which manifestly aligns with increasing trends, Instagram empowers consumers to determine media content, rather than paid experts, to be primarily distributed on the Internet (Holliman, 2014; Hollebeek and Macky, 2019).

The content farm employs freelancers, including bloggers and part-time writers, to produce content on trending topics, resulting in a high search and browse traffic to the websites (Bakker, 2012). Being online, however, means the presence of duplicators, as free content can be assessed, copied, and republished by others effortlessly. This situation is now occurring on Instagram as well. Some users possess several accounts related to different hot areas to raise their income by attracting various audiences. However, they cannot manage every account well by posting 5–6 quality photos every day. So, they purchase photos with a caption from a part-time photo designer. Usually, the pictures in this transaction are low quality, useless, or even copied from other accounts. Low-quality content cannot build purchase intention, even if it hits the trend and favor of the audience. Nevertheless, content marketing can deliver valuable information (Bakker, 2012).

One of the traditional digital marketing campaigns is paid advertisement, which includes pop-up and embedded ads in a website and search engine, as well as intersection commercials before and during videos. On Instagram, paid advertisements will appear as intrusive advertisements on Instagram stories and on the home page. As mentioned in the research of Li et al. (2002), intrusiveness occurs when commercials disturb the ongoing entertaining activities of the user. Forced and intrusive businesses will injure consumer perceived value, and the consumer may even respond negatively. Diversely, the perceived intrusiveness level of an advertisement will be decreased when the user finds the content is valuable and consistent with the websites or editorials (Ying et al., 2009). Paid advertisements finally decrease purchase intention (Goodrich et al., 2015).

This section will be described from the digital marketing to SMM and DCM, as DCM is in the subset of digital marketing, and adopting DCM on Instagram is one of the SMM methods. The attributes and advantages of DCM are also captured from the literature. Some popular content marketing frameworks are included to illustrate how to develop an effective DCM and why DCM can have those benefits.

### Digital Content Marketing

Digital Content Marketing refers to the act of conducting all marketing-related activities through the Internet, including advertising, purchasing process, customer service, and delivery service (Koiso-Kanttila, 2004; Ahmad et al., 2016; Naidoo and Hollebeek, 2016). Holliman (2014) proposed that inbound marketing is more efficient and effective in costing, spreading, extending the customer boundary, and co-creating value. DCM, which delivers valuable and interactive content to potential customers, is a technique to support inbound marketing.

Instead of putting significant effort and resources into outreaching leads, DCM focuses on creating excellent content that can provide a long-tail effect (Ahmad et al., 2016; Hollebeek and Macky, 2019). People describe DCM as an art of communicating with the customer, but without directly selling a product (Koiso-Kanttila, 2004; Ahmad et al., 2016; Hollebeek and Macky, 2019). By creating great content and e-conversation, a company can build a relationship with its existing customers, acquire new customers, retain customer loyalty, and build a reliable brand name. Moreover, the company can cultivate sales activity through customer engagement, loyalty, and relationship in the long run (Holliman, 2014). Killing content has the features of all branded content, random content, and the content the customer wants to know. Therefore, it is interestingly relevant to the customer engaging and syndicating. DCM is able to share valuable and free content related to the brand or the field. Moreover, DCM can attract and convert audiences to customers and repeat consumers (Le, 2013). People come to read, see, learn, and experience; therefore, the company usually tells its unique and meaningful stories to grasp and retain customers’ attention, which also comes within DCM’s scope and as a particular content form of company images (Holliman, 2014). Pulizzi and Barrett (2009) explained that companies are the experts in their business fields, and they can capture the most reliable and latest content resources. Therefore, a lead interested in the content may be willing to search or investigate certain content. Expert content can draw the attention of potential customers, who can understand the value of the content and product provided (Stone and Woodcock, 2014).

By creating great content and customer engagement, a company can build brand awareness, acquire new customers, retain customer loyalty, and finally achieve repeat sales (Holliman, 2014; Hollebeek and Macky, 2019). The customers have a more favorable attitude and confidence toward the product and the brand, as they are the hot trend between peers. It can raise the trust of online sellers, and the OPI can be boosted (Hajli, 2014). The brand, adopting DCM strategies, is discovered by customers when they demand the relevant content or product, thereby revealing a more significant consumer-engaged attitude. It is different from intrusive advertisements, which interrupts current activity while delivering a sales message (Holliman, 2014; Hollebeek and Macky, 2019). Through direct conversation and continuous interaction, sellers can engage their customers who are willing to buy. Sellers can also suggest appropriate products to customers to make purchase decisions collaboratively (Chen et al., 2011). Moreover, quality content helps maintain customer loyalty with two-way conversation (Koiso-Kanttila, 2004; Ahmad et al., 2016; Naidoo and Hollebeek, 2016).

**Figure 1:**
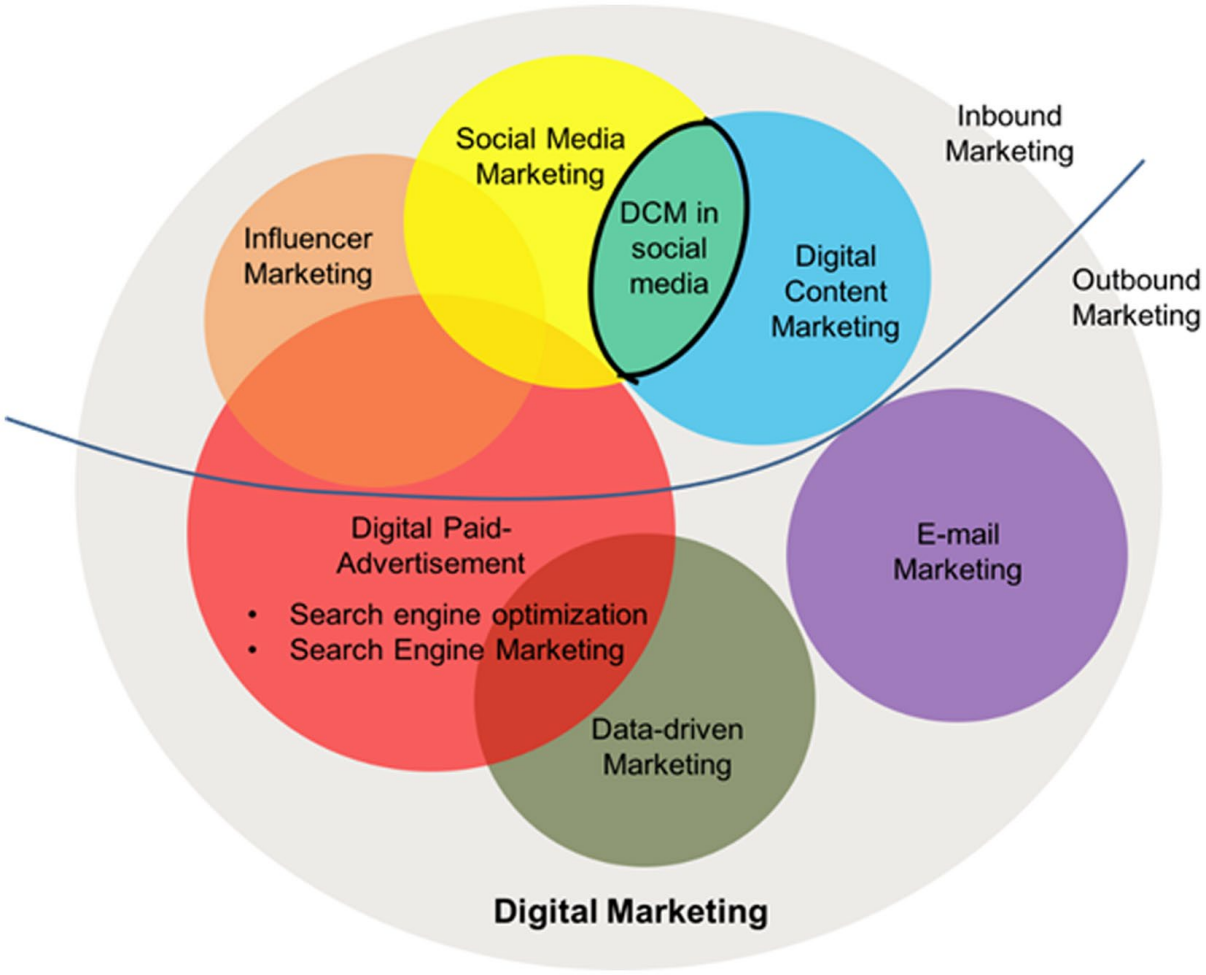
A conceptual framework of digital marketing.

As shown in Figure 1, adopting DCM on social media is a part of Social Network Marketing (SNM), and companies not only grasp the opportunity, the emerging trends of SNM, to cultivate long-term OPI, but also boost immediate OPI by delivering better content (Madsen and Slåtten, 2015; Ahmad et al., 2016; Wertalik, 2017; Mason et al., 2021b; Abbas et al., 2022). The current literature has been found to describe the attributes and advantages of DCM, and numerous empirical studies have been conducted to find the influences of SNM on OPI (Yang et al., 2016; Bolat and O’Sullivan, 2017). Research on the impact of Social Media Content Marketing (SMCM) on brand health indicated that SMCM plays a vital role in brand health since it acts as the inter-connection for a potential customer to grasp the brand’s information (Canhoto et al., 2015; Ahmad et al., 2016; Kareem et al., 2016; Mason et al., 2021a). However, no study has investigated the effectiveness of DCM on both immediate and long-term OPI simultaneously. Conducting DCM on social media is an SNM strategy, but not every marketing in social media can be concluded as DCM. Thus, the influences of DCM *via* social media may not be fully equal to SNM, which may be affected by more attributes (Dessart, 2017; Valos et al., 2017; Tafesse and Wien, 2018). For example, the familiarity and the perceived value of a product. This study is an empirical investigation to evaluate the effectiveness of DCM on both immediate and long-term OPI. A conceptual model based on the extant literature is developed to evaluate the DCM’s efficacy with SEM.

### Immediate Purchase Intension

In the research investigated by Chinomona et al. (2013), the significant positive impact of perceived product quality on perceived product value was investigated. The customer’s perceived value of the product can affect OPI. The researchers proved that the interviewees perceived the product as valuable when they recognized that the product had good quality and excellent attributes. The perceived product quality is about cognitive familiarity based on impressions, advertisements, and comments from others on the product. Therefore, OPI can reduce the perceived risk and increase transaction intentions (Chinomona et al., 2013). Hollebeek and Macky (2019) believed that content marketing is customer-oriented, as it aims to offer suitable solutions to customers with persuasive argumentation and helps customers to recognize the outstanding advantage of the product before they buy it, but not force them to buy (Koiso-Kanttila, 2004; Rowley, 2008; Holliman, 2014). The valuable content can comfort customers by telling them how the product/service meets their demands and what they can gain from it.

*H1*: DCM under MR environment on social media is positively related to the perceived value on the product/service.

Familiarizing with the perceived value can build trust in the product, and a higher level of trust can unlock customer’ OPI. It has been proven that the product’s perceived value can reinforce the immediate OPI through trust, reducing consumer perceived risk (Chinomona et al., 2013). Therefore, the perceived value of OPI can be immediately obtained, when customers receive the engaging content that entices people to take some kind of action. In addition, branded content, that combining both advertising and entertainment into one marketing communication content, could link to organization brand.

*H2*: Perceived value on the product/service is positively related to the immediate OPI.

### Long-Term Purchase Intention

Social Network Marketing adopts social media as a platform for brands to interact with their customers to develop further purchaser relationships, which helps to maintain loyalty and repeat purchase (Meyer-Waarden and Benavent, 2006; Wu et al., 2008; Papagiannidis et al., 2013; Malthouse et al., 2016; Nabec et al., 2016). Adopting DCM on social media, the seller can deliver quality content in different media types (text/audio/photo/video) on the post, story, and profile (Ahmad et al., 2016). The bargaining power in the market has shifted from sellers to buyers through the capability of the Internet, which significantly leverages the consumer’s voice (Mohamad et al., 2018). Companies can no longer make a unilateral decision regarding the price, quality, and after-sales service, and are being pushed to participate in conversations with customers to understand their needs and cultivate a close relationship through customer engagement. Therefore, excellent customer engagement can be cultivated by adopting DCM on social media, as SNM has been proven to have a significant influence on customer engagement (Areeba et al., 2017; Mohamad et al., 2018; Rozina et al., 2019; Hartiwi et al., 2020).

Brand trust indicates that consumers feel comfortable and are willing to make OPI, even in a situation of uncertainty (Laroche et al., 2012). Enduring involvement with the product has been demonstrated to positively influence brand trust (Erik, 2019). The enduring content allows customers to get familiarized with the product and the brand communities. Customers, therefore, cultivate more brand trust as they have perceived less risk and reduced uncertainty (Laroche et al., 2012). Especially for new leads, initial trust is formed through the brand impression by the available information of the product and brand communities, which is the critical element that DCM will deliver (Stouthuysen et al., 2018).

*H3*: DCM under MR environment on social media is positively related to customer engagement.

*H4*: DCM under MR environment on social media is positively affecting brand trust.

Enduring conversation and excellent customer engagement can increase familiarity between the seller and customer. As mentioned, the seller, or the content provider, is the expert in the specific area related to the content. Excellent content can build trust between the seller and customer, as the customer will perceive it worthwhile and reliable if they can absorb valuable knowledge during online shopping. Ahmad et al. (2016) found that sites will lose their customer’s interest if they only deliver simple responses or quick answers to their customer’s inquiries. Differing from that, social media sites, which provide unique content with plentiful customer engagement, can gain more customer attention and trust.

Either cognitive, emotional, or behavioral customer engagement acts as the primary effect (first-tier) of DCM, and intra-interaction, respectively, fosters the brand-related sense-marketing, citizenship behavior, and identification (second-tier) through different customer engagement. The third tier consequence of DCM is trust, either on credibility or benevolence and brand attitude. DCM will finally affect consumer-based brand equity and firm-based brand equity (Hollebeek and Macky, 2019).

Functional motive can also integrate with the hedonic motive (for example, entertaining and interesting content) to drive behavioral engagement, which means customers are willing to spend time, effort, and energy interacting with the brand. Moreover, functional motive integrates with authenticity motive (integrity and credibility content) and can cultivate cognitive engagement, and authenticity motive combines with hedonic involvement to achieve emotional engagement. Besides spending effort on interaction with the brand, people are triggered into brand identification and sense of belonging and further achieve trust on either credibility or benevolence (Hollebeek and Macky, 2019).

*H5*: Customer engagement is positively related to brand trust.

Besides attracting more leads, detailed and trusted content can obtain higher customer retention (Pulizzi and Barrett, 2009), which means the seller will be the priority choice. Moreover, social media have been proven to play a significant role in OPI, as trust can be cultivated and accumulated through quality product/service, customer engagement, and trust (Mohamad et al., 2018). Areeba et al. (2017) has pointed out that customer engagement becomes a primary concern for the online retailer as the accumulated emotional ties between customers and companies help to convince their consumers to make the right buying decision (Rose and Samouel, 2009; Hollebeek, 2011; Leckie et al., 2016; Dessart, 2017).

**Figure 2:**
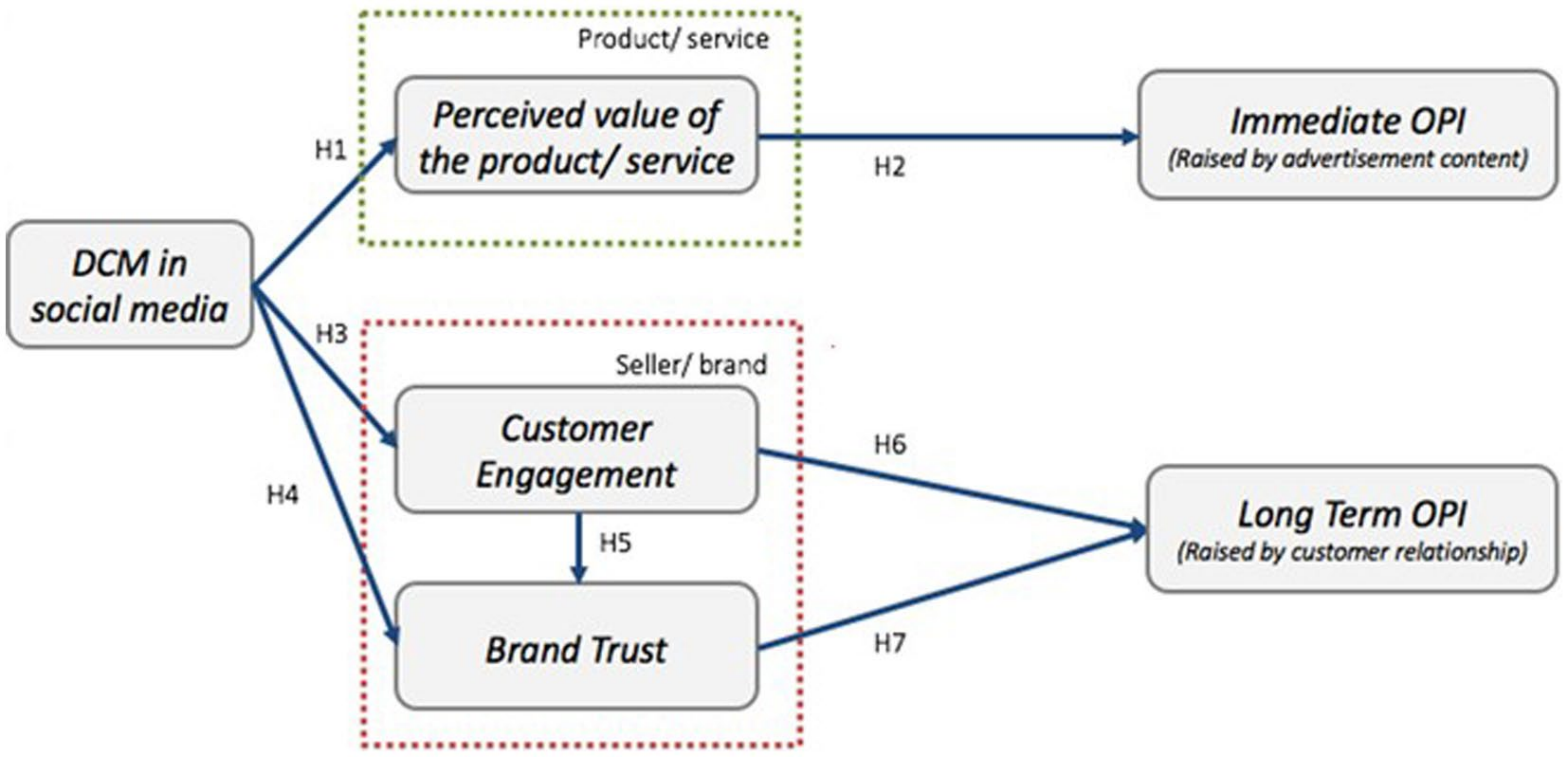
Conceptual model: digital content marketing (DCM).

Hollebeek and Macky (2019) illustrated three incentives that can drive customers to make purchase decisions through interaction with DCM communications. For the functional motive, customers are willing to seek valuable information. Regarding the hedonic motive, customers found that they can entertain themselves, relax, and absorb the knowledge if they are enjoying the content. Regarding the authenticity motive, the ultimate desires of consumers are achieved through the brand-related connection, integrity, credibility, and customer relationship from DCM (Hollebeek and Macky, 2019). Rather than persuading potential customers to purchase the product directly, DCM is designed to develop and reinforce consumer engagement, awareness, trust, and the relationship between both parties (Ahmad et al., 2016; Hollebeek and Macky, 2019). Therefore, DCM can increase long-term sales and lead to repeat sales by accumulating relationships and trust. Figure 2 shows the conceptual model of DCM.

*H6*: Customer engagement is positively related to long-term OPI.

*H7*: Brand trust is positively related to long-term OPI.

## Methodology

A survey was conducted to obtain the opinion and perception of the effect of DCM under an MR-based training platform environment among Hong Kong residents. The effect of DCM on OPI was analyzed by SEM. Around 1000 questionnaires were distributed to the participants through Google Form from November 2021 to January 2022. Only the digital surveying method has been adopted in the research due to the outbreak of COVID-2019.

Mobile MR-apps enhance retail visits, including online shopping adoptability, by providing multiple product demonstration capabilities (Alcañiz et al., 2019; Wedel et al., 2020; Castillo and Bigne, 2021). The questionnaire presented the DCM simulating the online purchase intention through Instagram’s social media. The DCM is designed for multiple scenarios, including online travel agencies, fashion, beauty, and electronic products, shown in an MR on mobile devices. The research had two sections. In the first part, the participants used the mobile device to conduct three template scenarios, including online travel agencies, fashion, beauty, and electronic products with Instagram. Those contents are designed under the MR-based mobile environment. All the participants also conducted MR-based digital content through social media as examples for simulating the effects of the real-world DCM through social media. The second part consisted of an online questionnaire that assessed six constructs based on literature.

There are five reasons for choosing Instagram rather than another social media and digital platform. First, the user base of Instagram has been observed a significant growth trend, which is more evident than others. Second, the target groups of online retail in Hong Kong are mainly teenagers, young people, and middle-aged people, which entirely match the majority of user groups of Instagram. Third, as social media marketing simultaneously, DCM on Instagram can seize its benefits, for instance, customer engagement, user-generated content, and electronic word-of-mouth. Moreover, Instagram has many functions for corporations to communicate with audiences, and the graphics-based peculiarities allow them to deliver engaging and valuable content to their fans efficiently. Last but not least, there are many e-commerce and boutiques on Instagram. Sellers can market their customer and audiences simultaneously. Therefore, the search is to determine the effectiveness of DCM on Instagram. The experimental subjects are active users of social media in Hong Kong.

The questionnaire was designed based on Confirmatory Factor Analysis and a multi-item measurement scale. A seven-point Likert-type scale, where one indicates “strongly disagree” and seven interprets “strongly agree,” was adopted to evaluate the perception on different dimensions related to DCM (Park et al., 2004; Leong et al., 2015). The questionnaire items are summarized and modified based on the literature whereas the hypothesis settings are. Hence, the questionnaire included six constructs, measured on various scales adapted from previous studies.

The online survey was designed for Hong Kong citizens who use social media frequently. The participants were voluntary anonymous, and the results were confidential. The survey was first developed in English based on the literature and previous studies and translated into Chinese by a bilingual researcher. Three questions have been asked to indicate the Chinese and English proficiency level and any language-related difficulties.

Two screening questions developed that the potential participants were regular social media users and online shopping customers. Only participants who were regular social media users and online shopping customers were considered. Five incomplete responses answered by people who do not use any social media were found, nor non-online shopping users, and 25 invalid responses failed to answer Neutral in either one or both verification questions. There were 374 questionnaires in total qualified for the data analysis in this study. The model fit indices are affected by the sample size significantly. Therefore, Boomsma and Hoogland (2001) recommended that CFA have more than 400 samples and at least a sample size of *N* > 200 (Lee et al., 2018a).

## Data Analysis and Results

The SEM was analyzed with IBM SPSS Statistics 25 and IBM SPSS AMOS 25, ensuring reliability, confidentiality, and significance level. As a method for the covariance-based SEM, the AMOS provides more flexibility for data requirements. The benefit of AMOS-SEM is that it offers a parameter estimation model assessment and is fit for use in reflective indicators and parameter estimation modeling. Hence, AMOS-SEM performs well even when the sample size is large compared to, e.g., PLS-SEM.

### Respondents’ Characteristics

Respondents’ characteristics are reported in Table 1. The gender distribution of the qualified surveys is 178 Male (47.59%) and 196 (52.40%) Female. Nearly half of the participants fell into the 19–24 age group, followed by the 25–34 age group (35.56%) and the 35–44 age group (8.56%). Around 357 out of 374 participants had or were pursuing an Associate Degree/Higher Diploma or Bachelor’s Degree or above. The respondents’ characteristics also include the times of online shopping in the past 12 months, using social media habits, adopting different social media platforms, and reasons for online shopping. More than 75% of the respondents use social media for more than 2 h per day. Facebook, Instagram, and YouTube are the top priority social media usage in Hong Kong, of which nearly 90% of the respondents have been using. More than 75% of respondents have been shopping online during the past 12 months. The significant reasons for shopping online are convenience and a wide selection of choices. Therefore, the questionnaire results can show the opinions of significant online consumers in Hong Kong.

**Table 1:** Respondents’ characteristics.

Attributes	Total sample (*n* = 374)	
	Frequent	Percentage
Gender		
Male	178	47.59%
Female	196	52.40%
Age		
Below 18	6	1.60%
19–24	184	49.20%
25–34	133	35.56%
35–44	32	8.56%
45–54	13	3.48%
Above 55	6	1.60%
Highest education level (/pursuing)		
Primary school or below	1	0.27%
Secondary school	14	3.74%
Associate degree/higher diploma	189	50.53%
Bachelor degree or above	168	44.92%
Prefer not to say	2	0.53%
Using habit of social media		
Using monthly	3	0.80%
Using weekly	3	0.80%
Using daily but less than 2 h a day in average	81	21.66%
More than 2 h a day in average	287	76.73%
Social media platform (can choose multiple answer)		
Facebook	331	88.50%
Instagram	367	98.13%
YouTube	362	96.79%
WeChat moments	138	36.90%
Twitter	121	32.35%
Snapchat	82	21.92%
LinkedIn	56	14.97%
Pinterest	22	5.88%
TikTok	121	32.35%
Weibo	17	4.54%
Times of online shopping in the past 12 months		
Never	12	3.21%
One time	13	3.48%
2–4 times	70	18.71%
5–10 times	217	58.02%
11 times or above	62	16.58%
Reasons of often online shopping (can choose multiple answer)		
Convenience	370	98.93%
No crowds and queues	187	50.00%
Competitive price	260	69.52%
Wide selection of choices	301	80.48%
Free returns or exchanges	68	18.18%
Easy to compare price	247	66.04%
Can refer to others’ comments and reviews	224	59.89%
The online shopping platform is easy to use	150	40.11%
The online store is almost never closed	94	25.13%
The online store is trustworthy	57	9.89%
The product I can get from online shop only	33	8.82%

### Measurement Model

Given that the results were similar, only the sample results as a whole are presented. Hair (2010) suggested convergent validity and the measurement reliability of data should be assessed by Standardized factor loading $\left(K\right)$ of each measurement items, Cronbach’s alpha $\left(\alpha \right)$ , Composite reliability (CR), and Average Variance Extracted (AVE). Convergent validity measures of constructs that theoretically are related to each other are, in fact, observed to be related to each other. The value criteria, which indicates that the data are reliable and valid, are shown as follows: K is excellent when greater than 0.7, good between 0.5–0.7; α should be greater than 0.7 (Freeze and Raschke, 2007; Corrêa et al., 2020). Bagozzi and Yi (2011) suggested C.R. should be higher than the acceptable levels of 0.700; AVE should be greater than 0.500, or 0.400 in the cases of exploratory research (Corrêa et al., 2020). The majority of factors show sufficient internal consistency. Most of the measurement items’ K were above 0.7, and at least over 0.6. The α of the constructs were above 0.7, and ranged between 0.761 and 0.863. The C.R. varied between 0.717 and 0.833 and AVE loaded between 0.499 and 0.621. Therefore, the majority of measurements had significant internal consistency, and a few measures had relatively low reliability but also supported the convergent validity. Table 2 summarizes the confirmatory factor analysis, which includes K , α , C.R., and AVE on the constructs and measurement items. Table 2 also lists the questionnaire items which are based on a certain of literatures. Discriminant validity was tested using item cross-loadings, which indicates that a construct should share more variance with its indicators than with other constructs (Castillo and Bigne, 2021) shown in Table 3. The value of the correlations was significant value of *p* < 0.01, except for brand trust and customer engagement.

**Table 2:** The measurement model (convergent validity).

Constructs items (reflective)	Number of items	Number of items deleted	*K*	*α* > 0.7	*e*	C.R. > 0.7	AVE > 0.5
DCM in social media Koiso-Kanttila, 2004; Rowley, 2008; Holliman, 2014; Ahmad et al., 2016; Hollebeek and Macky, 2019	3	0		0.761		0.743	0.502
DCM1: DCM under MR environment provides enough details and information about the Product/Service (e.g., the materials of the product/the functions of the product/some ideas to better utilize the product).			0.765		0.58		
DCM2: The Product/Service described in DCM under MR environment is attractive.			0.751		0.56		
DCM3: DCM under MR environment is relatively less intrusive than the paid-advertisement marketing campaign.			0.821		0.27		
Perceived value of the Product/Service Chinomona et al., 2013	3	0		0.863		0.749	0.503
V1: I can perceive a great value of the Product/Service described in DCM.			0.835		0.72		
V2: It is worth the price to have the Product/Service described in DCM under MR environment.			0.812		0.66		
V3: The description in DCM under MR environment let me realized that the Product/Service can cater to my needs.			0.873		0.76		
Immediate OPI Koiso-Kanttila, 2004; Rowley, 2008; Holliman, 2014	5	0		0.834		0.833	0.621
IPI 1: I want to buy the Product/Service because I found it has powerful features.			0.731		0.53		
IPI 2: The more I know the Product/Service, the more OPI on it.			0.671		0.44		
IPI 3: I want to buy the Product/Service because I believe I can make good use of it to improve my living quality.			0.702		0.49		
IPI 4: I want to buy the Product/Service because the excellent quality described in DCM.			0.773		0.59		
IPI 5: I want to buy the Product/Service because I believe it can create great value.			0.773		0.59		
Customer engagement Hollebeek and Macky, 2019	3	0		0.862		0.717	0.499
CE1: DCM under MR environment is interactive that the communication between me and the company is bilateral.			0.861		0.73		
CE2: I have different ways to contact the companies/sellers, which adopted DCM under MR environment, either like, comment, direct message, story interaction, or hashtags in social media.			0.892		0.77		
CE3: I have positive customer experiences as I can get assistance in time.			0.791		0.83		
Trust on seller Hollebeek and Macky, 2019	4	0		0.840		0.803	0.505
T1: More communication with the editor can leverage the trust on the company.			0.761		0.58		
T2: I can gain more Brand Trust by reviewing the comments from other users.			0.753		0.56		
T3: The continuous interaction makes me believe the company is trustworthy and reliable.			0.774		0.59		
T4: I believe that more customer engagement interprets the company cares what its customer wants so that they can offer a better and suitable Product/Service.			0.771		0.55		
Long-term OPI Ahmad et al., 2016; Hollebeek and Macky, 2019	4	0		0.829		0.798	0.501
LPI 1: I will be at ease if the company cares about their followers, for example: gives a response to any enquires in time.			0.852		0.73		
LPI 2: The company is reliable if the company tackles the customer’s problem reasonably.					0.811				0.65		
LPI 3: I will shop online if the seller gains positive comments from other users.					0.753				0.58		
LPI 4: The reliable seller can leverage my OPI.					0.632				0.39		

**Table 3:** Discriminant validity (correlations between constructs).

Latent constructs	DCM	Perceived value	Customer engagement	Brand trust	Immediate OPI	Long-term OPI
DCM	0.709					
Perceived value	0.620	0.709				
Customer engagement	0.486	0.629	0.788			
Brand trust	0.414	0.604	0.635	0.706		
Immediate OPI	0.458	0.535	0.778	0.628	0.710	
Long-term OPI	0.527	0.528	0.538	0.632	0.578	0.708

These two formulas calculate the C.R. and AVE:


$$C.R.=\frac{\sum \left(K^{2}\right)}{\sum \left(K^{2}\right)+\left(\sum e\right)}$$

$$AVE=\frac{{\left(\sum K\right)}^{2}}{{\left(\sum K\right)}^{2}+\left(\sum e\right)}$$

e = residual/error

To examine the common method bias, Podsakoff et al. (2003) proposed and summarized for the confirmatory factor analysis was estimated, restricting all the indicators in the model to load on a single factor. Table 4 shows the model absolute fit measures. The Goodness-of-fit index (GFI) is adequate when larger than 0.9, and a perfect fit with the value near 1.0 (Bentler, 1990). GFI scores in the range of 0.8–0.9 represent a good fit as they are quite affected by the sample size (Doll et al., 1994). Adjusted Goodness-of-fit index (AGFI) is further analysis from GFI considering the degree of freedom which is adequate when larger than 0.9 (Bentler, 1982). Standardized root means square residual (SRMR) scores less than 0.05 represent a reasonable (Jöreskog and Sörbom, 1989). Root Mean Square Error of Approximation (RMSEA) is recommended to be equal to/below 0.08 (Hair, 2010). Table 5 shows the model comparison fit measures. Normed fit index (NFI) values range between 0 and 1, and the higher value indicates a better fit (Ullman, 2001). NFI should be greater than 0.95, which is reasonable. Bentler (1990) and Schumacker and Lomax (2004) proposed that the value of NFI over 0.8 is acceptable, as it will be under loaded when analyzing with the small sample size. The non-normed fit index (NNFI) or The Tucker-Lewis Index (TLI) should be greater than 0.9 (Bentler and Bonett, 1980; Hoyle, 1995). Relative fix index (RFI) is the extension from NFI and should be greater than 0.9 (Bentler and Bonett, 1980). The comparative fit index (CFI) is similar to NFI but considers penalties. The value is typically greater than 0.9 (Bentler and Bonett, 1980). Table 6 shows the model parsimonious fit measures. Hair (2010) mentioned that *X*^2^ distribution should be less than 3 but greater than 1 would be the best scenario. Parsimonious goodness-fit-index (PGFI) and Parsimonious normed fit index (PNFI) should be greater than 0.5 (Bentler and Bonett, 1980). The results showed that the computed fit indices provided strong support for the hypothesis (GFI = 0.901; AGFI = 0.912; SRMR = 0.042; RMSEA = 0.031; NFI = 0.907; NNFI = 0.905; RFI = 0.911; CFI = 0.921; *X*^2^/df = 1.777; PGFI = 0.676; and PNFI = 0.741.).

**Table 4:** Model absolute fit measures.

Model fit	GFI	AGFI	SRMR	RMSEA
	0.901	0.912	0.042	0.031

**Table 5:** Model comparison fit measures.

Model fit	NFI	NNFI	RFI	CFI
	0.907	0.905	0.911	0.921

**Table 6:** Model parsimonious fit measures.

Model fit	*x*^2^/df	PGFI	PNFI
	1.777	0.676	0.741

**Figure 3:**
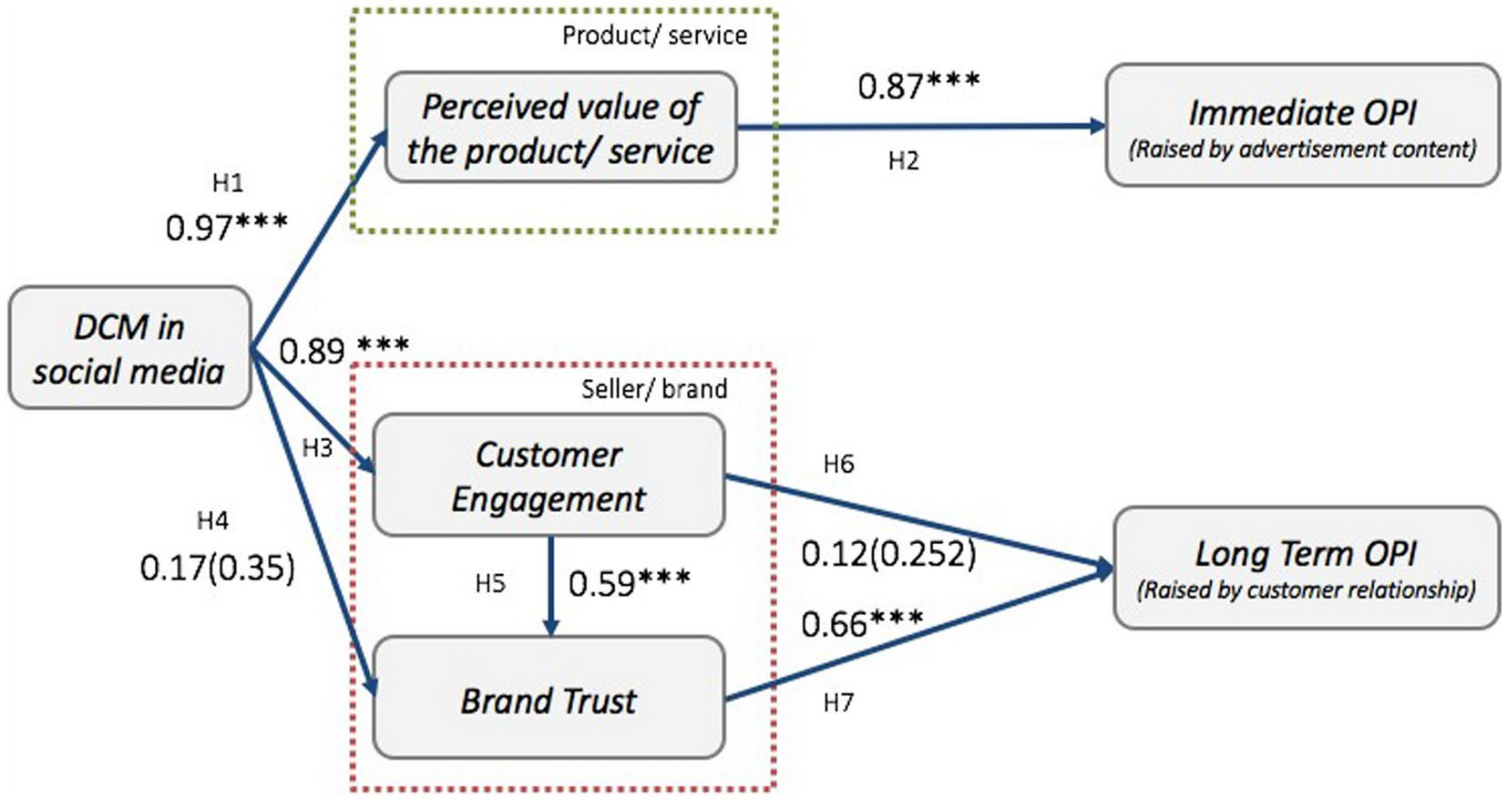
Structural Equation Model (SEM) result. ***< 0.01.

The proposed model was evaluated, and the estimated path coefficient and *p*-value are presented in Figure 3. Table 7 summarizes the hypothesis results of each measure. According to the result, Hypotheses H1, H2, H3, H5, and H7 are accepted, while H4 and H6 are rejected in the proposed model. DCM in social media is strictly related to both the perceived value of the product/service H1: *β* = 0.97, *p* < 0.01 and customer engagement H3: *β* = 0.89, *p* < 0.01. The perceived value of the product/service stipulated a significant positive relationship with immediate OPI (H2: *β* = 0.87, *p* < 0.01). Customer engagement indicated a strictly positive relationship with brand trust H5: *β* = 0.59, *p* < 0.01. And brand trust significantly affects long-term OPI H7: *β* = 0.66, *p* < 0.01. Although the result does not point to a direct positive relationship between DCM and brand trust, exceptional customer engagement can reinforce brand trust. The result illustrates that customer engagement has no significant direct effect on long-term OPI, while customer engagement still affects OPI through increasing brand trust. Table 8 shows the mediating effects which standardized indirect effects of mediators. As a result, perceived value partially mediated the relationship between DCM and immediate OPI. Brand trust has partially mediated the relationship between customer engagement and the long-term OPI.

**Table 7:** Summary of the hypothesis testing results.

Hypothesis	Path	*β*	Sign.	*t*-value	Result
H1	DCM → Perceived value	0.97	<0.01	14.842	**Accepted**
H2	Perceived value → Immediate OPI	0.87	<0.01	9.503	**Accepted**
H3	DCM → Customer engagement	0.89	<0.01	13.146	**Accepted**
H4	DCM → Brand trust	0.17	0.35	0.935	Rejected
H5	Customer engagement → Brand trust	0.59	<0.01	3.128	**Accepted**
H6	Customer engagement → Long-term OPI	0.12	0.252	1.146	Rejected
H7	Brand trust → Long-term OPI	0.66	<0.01	5.685	**Accepted**

**Table 8:** The mediation impact.

Hypothesis (indirect effect) path	Path coefficient	Result
DCM → Perceived value → Immediate OPI	0.621^***^	Partial mediation
DCM → Customer engagement → Long-term OPI	0.462	/
DCM → Brand trust → Long-term OPI	0.567	/
Customer engagement → Brand trust → Long-term OPI	0.564^***^	Partial mediation

***< 0.01.

## Discussion

The findings confirm the assumption that good use of MR-based DCM could bring positive effect on both long-term and immediate OPI through mediating factors.

### Immediate OPI

Digital Content Marketing delivers practical, engaging, and correct content to its leads. Therefore, potential customers who have been receiving enough details about the product/service are willing to search for more details about the product. They can capture the characteristics and quality of the product, and it can obtain a high perceived value from the DCM under the MR environment marketing description (H1). People claim that they can realize whether the product/service can fulfill their demand, and whether the product/service is worth the price. One of the most critical online shopping intentions is the product’s quality and features. Consequently, more perceived value by customers will bring more behavioral OPI (H2) as they have an excellent perception of the product and perceive less risk of online shopping. Therefore, the immediate OPI can be cultivated, consistent with the SEM results from Chinomona et al. (2013).

### Long-Term OPI

Besides achieving immediate OPI (in terms of product/service) by delivering helpful content to the leads, DCM under MR environment can cultivate trust and customer loyalty by customer engagement, finally affecting long-term OPI positively. DCM under MR environment in social media can seize the benefits, for instance, customer engagement (H3), user-generated content, and electronic word-of-mouth. The respondents acknowledge that DCM in social media is interactive and can promote positive customer experiences. In this study, a strict relationship between DCM in social media with customer engagement was found, which is in line with the previous studies conducted by Areeba et al. (2017), Mohamad et al. (2018), Rozina et al. (2019), and Hartiwi et al. (2020).

Digital Content Marketing on social media was found to have no strictly positive effect on brand trust, while it affects customer engagement, which can finally boost brand trust (H5). Companies engage their leads with continuous interaction, and this presence helps them in times of trouble. In addition, leads will be provided with customized service and offered suitable and better product choices, as firms are more familiar with their leads and are able to recognize their desires. Thus, companies are recommended to develop brand trust through excellent customer experience and other users’ positive actions (likes or shares), as customers are perceived less risk and uncertainty (Laroche et al., 2012; Erik, 2019). The significant result between customer engagement and brand trust is in line with the conceptual framework developed by Hollebeek and Macky (2019) and the observation of Ahmad et al. (2016). As predicted, brand trust has a positive relationship with long-term OPI (H7), which is aligned with the previous study (Hwang and Zhang, 2018; Mohamad et al., 2018; Hollebeek and Macky, 2019). The participants claimed that they would have more OPI if the seller was reliable or gained positive comments from other users.

The absence of a direct positive relationship between DCM under MR environment and brand trust can be explained by the conceptual model of Hollebeek and Macky (2019). Under the model, customer engagement is the first-tier consequence of DCM, while brand trust is the third-tier consequence. A progressive relationship exists between DCM, customer engagement, and brand trust. Therefore, DCM can enforce brand trust, mediated by customer engagement. Although the result does not indicate the significant relationship between customer engagement and long-term OPI, which has been proved in various relevant studies, customer engagement accumulated brand trust and positively affected long-term OPI. The potential customer-generated per $1,000 spent by content marketing or paid search campaign was compared in the research of Le (2013). A paid search campaign can grasp the advantages as the company paid for the leads in the first one and a half years. However, leads from paid search campaigns are constant, but content marketing will have more rapid growth in the future because of the accumulated trust and loyalty. Content marketing can produce three times more than the paid search campaign in the last month of the third year (Le, 2013). The long-tail effect of DCM under an MR-based training platform will surprise everyone, as it requires time to acquire trust between both parties and occurs rampant growth. Therefore, there is no significant direct effect between customer engagement and long-term OPI, but a strict positive relationship between brand trust and long-term OPI exists. Thus, SMEs should not give up developing DCM, even if they cannot observe powerful results initially.

### Theoretical and Managerial Implications

It is no doubt that paid advertisements can reach many digital users who access the Internet through search engines, websites, social media advertisements, and video commercials on YouTube. However, they are intrusive and hard-selling and may result in annoying and negative impressions from leads, as they disturb the endless entertainment of the leads. Therefore, the viewers usually ignore the paid advertisements and close the paid advertisement page; some people even pay for the external blocker or subscribe to premium membership to avoid them (Truong and Simmons, 2010). Thus, paid advertisements are an expensive investment and lack effectiveness in recent years. With MR-enabled DCM, even SMEs can achieve extraordinary sales performance from their marketing campaigns and access their targeted customers with great content through two-way communication.

Social media networks are the most popular way people can grasp information. Launching the DCM in social media can present selling messages to their targeted customers effectively and avoid the issues of the traditional paid advertisements, for instance, intrusive marketing and misleading ads. With the feature of MR, customers may get to know more about the characteristics of a product or a service. The process itself also stimulates customers’ engagement with a brand. Li et al. (2002) pointed out that reducing intrusiveness has a significant positive impact on advertising effectiveness and customer engagement. E-commerce can capture customers’ preferences and massive data during the conversation and provide customized products (Pulizzi and Barrett, 2009). The longer the investment period of DCM, the more the substantial long-tail effect can be acquired (Le, 2013). Therefore, the Return on Investment goes up if the companies apply successful DCM, as they no longer need to spend on useless advertisements and related rent (Truong and Simmons, 2010).

Digitalization is a worldwide trend. Digital users in Hong Kong spend nearly 2.5 h on their mobile devices, an hour longer than they do on the TV. Last but not least, each person in Hong Kong had 2.3 devices and was enjoying 129.5 MB/s connection speed on average in 2017. 5G network technology has been launched, the access speed of digital content can be shortened to instant, and people can access various content more readily (Li et al., 2020, 2021a). With better bandwidth and lower latency, the MR scenarios that customers can experience would be attractive in further detail. All these figures showed that digital marketing in Hong Kong has tremendous potential to grow.

According to the marketing expenditure in Hong Kong, traditional advertising has been replaced by digital marketing since 2012 (Wong and Wei, 2018). The spending on the online advertisement has increased from 9% in 2012 to 32% in 2019. The total digital advertising value is now around 5.5 billion HKD (STATISTA, 2020). In the same period, TV advertising, which used to have the most market share, fell to 14% in 2019. The expected budget on digital marketing would reach 34% and be more than double TV ad spending by 2021. Digital marketing involves many varieties. Following the Hong Kong Digital Marketing Statistics, leads discover an unfamiliar brand through search engines the most (35%), followed by eWOM (29%), social media ads (24%), and recommendations on social media (21%; Kemp, 2019). To provide the best experience over the Internet, companies should put more resources into developing digital content marketing under the MR environment. This provides valuable and engaging content to raise immediate purchase intention and build trust and long-term purchase intention. Regarding the data analysis of SEM, the effectiveness of DCM on purchase intention in terms of both immediate effect and long-tail effect were proved either through familiarity with the product/service or customer engagement. The recommendations will focus on the three most popular industries in e-commerce: Fashion and Beauty, Airline and Travel, and Electronic Products. According to the study on online retail, more than 90% of respondents, who are frequent online shoppers, sometimes or always purchase clothes online, and over 50 and 35% of respondents buy books/toys and air ticket/travel online, respectively, from the Hong Kong Consumer Council Report.

Other frequently online purchase sectors are clothing and beauty, as the products are quickly replaced by trending items in the fast fashion industry. People are confident enough to purchase branded clothing even if they cannot physically inspect or try the items. They believe branded goods have passed quality assurance and they are comforted by the fact that they can exchange unwanted items. However, customers have low confidence toward unknown brands, which usually are SMEs. Regarding the reasons for never and rarely online shopping, around 50% of respondents claimed a lack of confidence in online shopping because they have had bad experiences before and could not physically inspect the product. A questionnaire has done by the consumer council shows that 22% of interviewees are afraid of online shopping and no confidence in the product quality. Lack of confidence will cause the purchase intention of the potential customer to collapse. However, online paid ads can reach many audiences but cannot cultivate their trust in the brand. Moreover, the companies should continuously invest a relatively large amount in promotion, as they have to pay for the marketing rent. Therefore, DCM on Instagram is a better approach for SMEs to achieve promotion goals with an affordable budget.

More and more people look for flight tickets, hotel booking, and travel tours through Online Travel Agent (OTA) rather than visit the physical travel agency. OTAs provide services 24/7 from anywhere, and users can compare the prices with several OTAs simultaneously rather than visit different physical stores. Expedia, Trip.com, Trivago, and Skyscanner are examples of famous OTAs. It is no longer attractive to promote tours only through paid advertisements on the search engine. Intrepid Travel is a travel agency, which mainly offers small groups, big adventures, and responsible travel. They have currently adopted DCM, showcasing aspirational travel images posted on Instagram and Facebook taken by real travelers, Intrepid Travel, is interspersing with its content. It also allows real travelers to share their experiences, which helps the company connect more with its core audience. Last but not least, Intrepid Travel shows its enthusiasm for travel by replying to comments, which can draw the connections with the viewers as both of them have share the same passion on the adventurous travel.

Moreover, the DCM approach offers solutions for companies to reach the target audience precisely, which means the companies can reach their ideal customers through social media. Although approaching a smaller group of leads, DCM allows sellers to focus on targeted customers, easily perceive the product’s value and have greater OPI. With the DCM assisted with MR, sellers have more valuable data collected by sufficient customer engagement to improve marketing insights. For example, the number of “likes” indicates how many people are interested in a product, and their comments may involve inquiries and attitudes to the product. Thus, the sellers can strengthen their marketing tactics according to online data. In addition, future fabrications can be adjusted following the trend and the preference of potential customers. Deeper interaction with the ideal customers can improve the behavior brand attitude and result in repeat purchases (Hollebeek and Macky, 2019). In particular, MR-based DCM has enormous potential to grasp a significant market share in the Hong Kong digital advertising market.

## Conclusion, Limitations, and Future Research

Regarding the result of the study, both the immediate and long-term OPI has been proved. The immediate impact comes from the perceived value toward the product or service described exhaustively in the DCM under the MR-based training platform environment. Furthermore, customer engagement can cultivate brand trust and enlarge the long-term OPI due to behavioral loyalty. The effectiveness of DCM under the MR environment has been introduced segmentally. However, it may take time to see the long-tail effect of DCM under the MR-based training platform, as the companies have to accumulate leads by continuously providing unique content. An effective marketing tactic for SMEs, DCM, a section of social media marketing, is suggested to take a significant component, supported by the paid advertising on either search engines or social media. MR can be further used and extended to enhance the customers’ experience and satisfaction. Online shopping in Hong Kong is most common among young and middle-aged adults and highly educated people, perfectly fitting the respondents’ characteristics. Therefore, the results can indicate the preferences and opinions on DCM for the above group of residents. However, online shopping market and e-commerce are proliferating, and people in other age groups and education levels may also be willing to accept and adopt the digital method of purchasing. The result will no longer be sufficient to represent all online shoppers. The findings fill the gaps in the literature by providing empirical evidence for OPI boosted by DCM *via* social media. Therefore, future research can be extended to broader respondents, who may have different responses and preferences on DCM. Future research could extend customer engagement and trust constructs with other individual difference variables and extend to the mediating effect on the antecedents. MR’s adaptability and effectiveness to different marketing channels could be further considered. The technology acceptance model and the theory of planned behavior model could be further analyzed for new model development. The multi-group analysis considering different countries could be considered. Consumer behavior under the MR-based platform for DCM could be a new construct to analyze further and consider.

## Data Availability Statement

The raw data supporting the conclusions of this article will be made available by the authors, without undue reservation.

## Author Contributions

CL, OC, and KK contributed to conceptualization. OC, YC, and KK performed data curation. YC and KK carried out formal analysis, performed investigation, and contributed to project administration. CL contributed to funding acquisition. OC, PT, and KK provided methodology. CL, OC, YC, and KK provided resources. PT and KK provided software. OC performed supervision. XZ, PT, and KK carried out validation. CL, OC, YC, XZ, PT, SL, HN, and KK helped with visualization. OC, YC, PT, and KK performed writing—original draft. CL, OC, YC, PT, and KK performed writing—review and editing. All authors have read and agreed to the published version of the manuscript. All the authors contributed to the article and approved the submitted version.

## Funding

The research was supported in part by the School of Science and Technology, Hong Kong Metropolitan University, Hong Kong SAR, China and in part by the Division of Business and Hospitality Management, College of Professional and Continuing Education, The Hong Kong Polytechnic University, Hong Kong SAR, China. The work described in this paper was partially supported by the grant from the Research Grants Council of the Hong Kong Special Administrative Region, China and Hong Kong Metropolitan University (Project No. R7016, Reference code: 2020/3003).

## Conflict of Interest

The authors declare that the research was conducted in the absence of any commercial or financial relationships that could be construed as a potential conflict of interest.

## Publisher’s Note

All claims expressed in this article are solely those of the authors and do not necessarily represent those of their affiliated organizations, or those of the publisher, the editors and the reviewers. Any product that may be evaluated in this article, or claim that may be made by its manufacturer, is not guaranteed or endorsed by the publisher.
